# Softening of the Brain

**Published:** 1859-01

**Authors:** 


### SOFTENING OF THE BRAIN

Is a disease for which there is no known remedy: its progress is slow, steady, and resistless as an avalanche, and body and mind go out together. It generally comes on with a gradual loss of sight, while the health of the remainder of the body is usually good. The younger son of the "Iron Duke" has recently died of this disease, which is becoming of more frequent occurrence than formerly. For eight long years he had been totally blind, and had amused himself with making willow baskets. It usually attacks men who have overworked their minds. But Lord Charles was neither a student nor a roué; but, being a man of great wealth, he lived at his ease. There were no sufficient inducements to mental and bodily activities—hence mental and physical stagnation first, then disorganization; and he died prematurely, in the midst of his millions.

Multitudes think it a hard necessity to tug and toil for daily bread, or that it should require their undivided energies of body and mind in planning and contriving and laboring to maintain their position. This is not a hard, but a happy necessity, as these very activities are not only the preservatives of body and mind, but are productive of those utilities which hasten human progress, develope our powers, elevate the people, and happify mankind.

